# Prevalence and prognosis of genetically proven familial hypercholesterolemia in subjects with coronary artery disease and reduced ejection fraction

**DOI:** 10.1038/s41598-023-44065-y

**Published:** 2023-10-07

**Authors:** Wen-Jane Lee, Han-Ni Chuang, Tzu-Hung Hsiao, Wen-Lieng Lee, Jen-Pey Wu, Wayne H.-H. Sheu, Kae-Woei Liang

**Affiliations:** 1 Department of Medical Research, Taichung Veterans General Hospital, Taichung, Taiwan; 2 Department of Social Work, Tung-Hai University, Taichung, Taiwan; 3 Department of Public Health, College of Medicine, Fu Jen Catholic University, New Taipei City, Taiwan; 4 Institute of Genomics and Bioinformatics, National Chung Hsing University, Taichung, Taiwan; 5 Cardiovascular Center, Taichung Veterans General Hospital, 1650 Taiwan Boulevard, Sec. 4, Taichung, 40705 Taiwan; 6 Department of Post-Baccalaureate Medicine, School of Medicine, National Chung Hsing University, Taichung, Taiwan; 7 School of Medicine, National Yang Ming Chiao Tung University, Taipei, Taiwan; 8 Institute of Molecular and Genomic Medicine, National Health Research Institutes, Miaoli, Taiwan; 9 School of Medicine, National Defense Medical Center, Taipei, Taiwan

**Keywords:** Genetics, Biomarkers, Cardiology, Medical research, Molecular medicine

## Abstract

Few studies have genetically screened variants related to familial hypercholesterolemia (FH) and investigated their survival impact in patients with coronary artery disease (CAD) and reduced left ventricular ejection fraction (EF). Patients with CAD and reduced EF (< 40%) were enrolled. Their genomic DNAs were sequenced for FH-related genes. All-cause and cardiovascular mortality data served as the major outcome. A total of 256 subjects were analyzed and 12 subjects (4.7%) carried FH-related genetic variants. After a median follow-up period of 44 months, 119 of the study subjects died. Cox survival analysis showed that carrying the FH genetic variant did not have a significant impact on the survival of CAD with reduced EF. However, higher estimated glomerular filtration rate (eGFR), better EF and beta blocker use were protective for a lower all-cause mortality. Further larger studies are needed to evaluate the impact of carrying the FH-related genetic variant on survival of CAD with reduced EF.

Subjects with coronary artery disease (CAD) have myocardial ischemia or infarction, as well as abnormal left ventricular ejection fraction (EF). Heart failure (HF) with reduced EF is associated with a nearly two-fold greater risk of 5-year mortality than those with preserved EF^1,2^. Familial hypercholesterolemia (FH) is caused by mutations in genes involved in cholesterol metabolism, resulting in impaired clearance of circulating low-density lipoprotein cholesterol (LDL-C). Growing evidence showed that most FH are the result of heterozygous pathogenic variants in three different genes that encode key proteins involved in the endocytic and recycling pathways, such as the LDL receptor (*LDLR*), apolipoprotein B (*APOB*) and proprotein convertase subtilisin kexin 9 (*PCSK9*)^3,4^. The prevalence of heterozygous FH in the general population of Caucasian is known to be approximately 1 in 250^3,5,6^, and 1 in 500 in Taiwan^7^. Subjects with FH are associated with an elevated risk of early-onset CAD^8–10^ and ischemic stroke^11,12^.

The prevalence of FH varies according to different clinical scenarios. A study showed that molecularly proven FH is present in 26.9% of patients in a cohort presenting with acute coronary syndrome and having LDL-C≧135.3 mg/dL^13^. Another study from Korea identified 10 variants in 10 patients (9.1%) from a population-based cohort of 110 subjects with total cholesterol levels ≧ 310 mg/dL^14^. In a study conducted in subjects with clinical suspicion of FH using Simon Broome criteria or LDL-C > 4.9 mmol/L with unknown family history, 52.1% of them had *LDLR* mutations and 4.2% had *APOB* mutations^15^. Genetically proven FH is present in 6.1% angiographically confirmed premature CAD^16^.

Regarding the risks conferred by FH, the SAFEHEART registry reported a more than 3 times prevalence of having angina pectoris, a 3.1-fold higher risk of acute myocardial infarction, and a tenfold chance of requiring coronary artery bypass surgery in the FH + group, compared with their unaffected relatives^17^. Patients with FH have higher rates of mortality (1.45-fold hazard ratio) and recurrent myocardial infarction (2.53-fold hazard ratio), after their first acute myocardial infarction compared to controls^18^.

Despite having studies on carriers of FH-related genetic variants in different clinical scenarios, few studies have comprehensively investigated the prevalence and prognostic impacts of genetically diagnosed FH on survival in patients with angiography proved CAD and reduced EF. Here, we aimed to genetically screen FH and determine its impact on all-cause or cardiovascular mortality in subjects with CAD and reduced EF based on a hospital catheterization laboratory cohort.

## Materials and methods

### Study population

**Figure 1:**
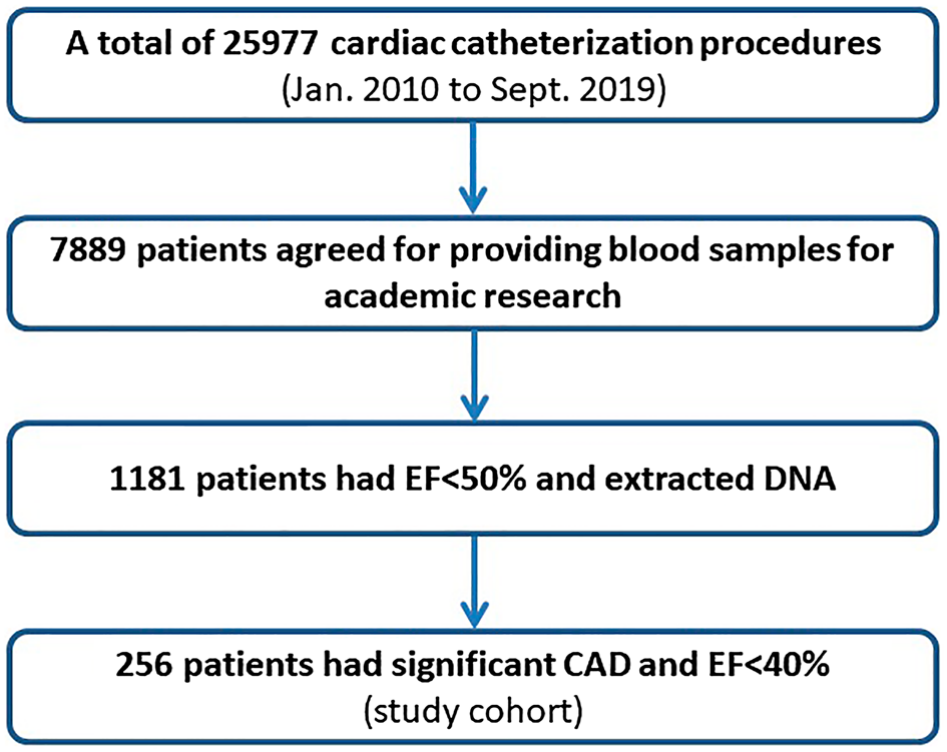
study enrollment protocol. *CAD* coronary artery disease, *EF* left ventricular ejection fraction.

The enrollment of subjects with CAD with EF < 40% has been fully described in our previous publications^19,20^. In summary, from January 2010 to September 2019, a total of 25,977 cardiac catheterization procedures were performed at our catheterization laboratories. Among them, 7889 patients agreed to donate blood samples for academic research on genetic, serums or plasma markers of cardiovascular diseases (Fig. 1). Among those 7889, 1181 had an EF lower than 50% and already had extracted DNA in stock. Subjects with significant CAD (SYNTAX score > 0^21^) or past histories of surgical or percutaneous coronary revascularization and EF lower than 40% were included for analysis (N = 256) (Fig. 1). Data recorded in the traceable medical chart records of this hospital included: the number of diseased coronary arteries, past histories of coronary revascularization by percutaneous coronary intervention (PCI) or coronary artery bypass graft (CABG) surgery, past histories of acute coronary syndrome, ischemic stroke, admissions for heart failure, peripheral vascular disease, atrial fibrillation, implantable cardioverter (ICD), and cardiac resynchronization therapy (CRT) and the medication history for CAD and HF. EF data were from echocardiograms obtained closest to the index admission. Our study protocol was approved by the Human Research Review Committee of Taichung Veterans General Hospital (Taichung, Taiwan). All methods were carried out in accordance with the relevant guidelines and regulations. Informed consent was obtained from all participants. All-cause and cardiovascular mortality were recorded until December 2019 and served as the main outcome. Mortality information was obtained from the Collaboration Center of Health Information Application, Department of Health, Executive Yuan, Taiwan.

### Definition of conventional risk factors for atherosclerosis

Hypertension was defined as systolic blood pressure > 140 mmHg or diastolic blood pressure > 90 mmHg based on multiple measurements at rest in the sitting position. Subjects with hypertension included those who had already received antihypertensive medication. Diabetes mellitus (DM) was defined as fasting blood sugar ≥ 126 mg/dl measured on two occasions. Subjects with diabetes mellitus included those who already received antidiabetic drugs or insulin injections. Serum creatinine was obtained at index admission for cardiac catheterization and study recruitment. The estimated glomerular filtration rate (eGFR) was calculated with the Modification of Diet in Renal Diseases (MDRD) equation^22^. Serum levels of triglycerides, cholesterol, and LDL-C levels were enzymatically using commercial kits (WAKO, Tokyo, Japan). Lipid profiles at the index admission (coronary angiography, blood DNA sampling, and informed consent) were used for comparisons as shown in Tables 2, 3 and 4.

### Genetic tests for familial hypercholesterolemia

Genomic DNAs were extracted from peripheral leukocytes using the QIAamp DNA Blood Mini kit (Qiagen, Hilden, Germany) for subsequent analysis with next-generation sequencing (NGS). Sequencing targets were for FH-related genes including whole exons of *LDLR, APOB, and PCSK9*. Probes/primers specific for these genes were designed and polymerase chain reactions (PCR) were performed to amplify the candidate DNA fragments prior to sequencing. Library construction was prepared using the QIAGEN target panel (QIAGEN, CDHS-15658z-227, Hilden, Germany). Each library so prepared was sequenced using paired-end runs on Illumina NovaSeq 6000 (San Diego, USA). The sequencing experiment was in accordance with the QIASeqTM Targeted DNA Panel Handbook. The FastQ files from Target DNA libraries were entered into the CLC Genomics Workbench 20 (QIAGEN, Demark), and reads of target sequencing were analyzed. The annotation of identified variants was implemented using Illumina’s Basespace Variant Interpreter (basespace.illumina.com)^10^. The pathogenicity assessment of the variants was evaluated with the Illumina Basespace Variant Interpreter and the 2015 guideline for molecular pathology of American College of Medical Genetics and Genomics (ACMG)^23^. Pathogenicity was further confirmed by the ClinVar database^24^. The ClinVar database is a public archive that provides information on human genomic variants with respect to their relationships with diseases and provides supporting evidence of clinical or functional significance^10,24^.

### Statistical analyses

Categorical data were expressed as percentage and compared using the Chi-square or Fisher’s exact test. Continuous variables were expressed as mean ± standard deviation and compared between groups using the independent t-test. Cox regression analysis was applied to determine independent predictors of all-cause or cardiovascular mortality in subjects with CAD and reduced EF. The SPSS (version, 25) statistical software package (SPSS, Inc., Chicago, IL, USA) was used for all calculations. A two-tailed p value of < 0.05 was considered statistically significant.

### Ethics approval

All procedures followed were in accordance with the ethical standards of the responsible committee on human experimentation (institutional and national) and with the Helsinki Declaration of 1975 as revised in 2000. The study was approved by the local ethics committee (log no. C09139B). All patients signed a written informed consent before inclusion in the study.

## Results

### Prevalence of carriers of FH-related genetic variant in subjects with CAD and reduced EF

In this study, we identified a total of 151 variants, including 6 pathogenic or likely pathogenic (in 12 patients, Table 1), 53 variants of uncertain significance (VUS), and 92 benign variants. A total of 12 subjects (12/256 = 4.7%) carried the pathogenic or likely pathogenic FH genetic variants (Table 1). All were heterozygous carriers. We detected two patients with *APOB* c.10579C > T missense mutation and one patient with *APOB* c.35_39del resulting in protein frameshift (Table 1). Nine subjects had variants of the *LDLR* gene, of which five were c.1747C > T missense (Table 1). We also reported their highest levels of LDL-C. Some variant carriers did not have a traceable record of LDL-C before statin use in this hospital (Table 1).

**Table 1:** Carriers of familial hypercholesterolemia related genetic variants in subjects with coronary artery disease and reduced left ventricular ejection fraction.

No	Sex	ID	Gene	Nucleotide change (cDNA)	Amino acid change	Variant type	Genotype	SNP	ACMG	Highest LDL-C record (mg/dL)*
1	M	V26A00072	*APOB*	c.10579C > T	p. (Arg3527Trp)	Missense	Het	rs144467873	Pathogenic	229
2	M	V26A00158	*LDLR*	c.1747C > T	p. (His583Tyr)	Missense	Het	rs730882109	Pathogenic	178
3	M	V26A00852	*LDLR*	c.190 + 4A > T		Splice region_variant, intron	Het	rs769446356	Pathogenic	150
4	F	V26A01774	*LDLR*	c.190 + 4A > T		Splice region_variant, intron	Het	rs769446356	Pathogenic	144*
5	M	V26A03166	*APOB*	c.10579C > T	p. (Arg3527Trp)	Missense	Het	rs144467873	Pathogenic	155
6	F	V26A03760	*LDLR*	c.811G > A	p. (Val271Ile)	Missense	Het	rs749220643	Likely pathogenic	176
7	M	V26A03795	*LDLR*	c.1747C > T	p. (His583Tyr)	Missense	Het	rs730882109	Pathogenic	193
8	M	V26A03916	*LDLR*	c.1747C > T	p. (His583Tyr)	Missense	Het	rs730882109	Pathogenic	130*
9	M	V26A04139	*LDLR*	c.769C > T	p. (Arg257Trp)	Missense	Het	rs200990725	Pathogenic	154
10	M	V26A04216	*LDLR*	c.1747C > T	p. (His583Tyr)	Missense	Het	rs730882109	Pathogenic	243
11	M	V26A04563	*APOB*	c.35_39del	p. (Leu12ProfsTer44)	Frameshift	Het	rs1664202070	Pathogenic	120*
12	M	V26A05585	*LDLR*	c.1747C > T	p. (His583Tyr)	Missense	Het	rs730882109	Pathogenic	239

Reference Sequences: *LDLR* NM_000527.4; *APOB* NM_000384.2.

*ACMG* 2015 The American College of Medical Genetics and Genomics guidelines, *Het* heterozygous, *APOB* gene encoding apolipoprotein B, *LDLR* gene encoding low-density lipoprotein receptor, *SNP* single nucleotide polymorphism, *LDL-C* low-density lipoprotein cholesterol data from available records.

*Some patients did not have a traceable LDL-C record before using lipid-lowering medication.

### Comparison of lipid profiles and clinical demographics in carriers of FH-related genetic variants vs. non-carriers in subjects with CAD and reduced EF

A total of 12 subjects (12/256 = 4.7%) carried the FH genetic variants (Tables 1, 2). Carriers of FH-reltaed genetic variants had a similar age and gender distribution as non-carriers (Table 2). The EF data and the number of coronary disease vessels were similar between carriers of FH-related genetic variants and non-carriers (Table 2). Carriers of FH genetic variants had a significantly higher serum total cholesterol level, LDL-C, and body mass index at index admission (Table 2). The number of disease vessels, the history of revascularization and history of medication were similar between carriers of FH genetic variant and non-carriers (Table 2).

**Table 2:** Subjects with coronary artery disease and a reduced ejection fraction (< 40%) (N = 256), who carried familial hypercholesterolemia related genetic variant (N = 12) or not (N = 244) (lipid data at the index admission).

	FH ( +) (N = 12)	FH ( −) (N = 244)	*p* value
Age (years)	66.8 ± 13.2	65.8 ± 13.2	0.815
Gender (M/F)	10/2	203/41	1.000
BMI (kg/m^2^)	28.5 ± 4.8	25.1 ± 3.9	0.006
DM N (%)	3 (25.0%)	124 (50.8%)	0.137
HT N (%)	9 (75.0%)	196 (80.3%)	0.711
Triglyceride (mg/dl)	191 ± 250	131 ± 101	0.427
Cholesterol (mg/dl)	203 ± 42	155 ± 36	< 0.001
LDL-C (mg/dl)	131 ± 33	93 ± 30	< 0.001
eGFR (ml/min/1.73 m^2^)	74 ± 21	58 ± 32	0.095
EF (%)	29.8 ± 7.5	29.9 ± 6.6	0.969
History			
ACS N (%)	5 (41.7%)	112 (45.9%)	1.0
Ischemic stroke N (%)	3 (25.0%)	39 (16.0%)	0.422
HF admission N (%)	9 (75%)	165 (67.6%)	0.757
Af	3 (25.0%)	49 (20.1%)	0.714
PAD	2 (16.7%)	49 (20.1%)	1.0
ICD	0	7 (2.9%)	1.0
CRT	0	11 (4.5%)	1.0
Disease vessel number			
1-VD N (%)	0	60 (24.6%)	0.110
2-VD N (%)	3 (25.0%)	63 (25.8%)	
3-VD N (%)	9 (75.0%)	121 (49.6%)	
Revascularization			
PCI N (%)	8 (66.7%)	188 (77.0%)	0.484
CABG N (%)	4 (33.3%)	73 (29.9%)	0.756
Medication			
Statin	8 (66.7%)	134 (54.9%)	0.556
ACEI/ARB	10 (83.3%)	193 (79.1%)	1.0
Beta blocker	6 (50.0%)	153 (62.7%)	0.379
ARNI	3 (25.0%)	35 (14.3%)	0.395
Ivabradine	1 (8.3%)	35 (14.3%)	1.0
Antiplatelet	12 (100%)	232 (95.1%)	1.0
PCSK9 inhibitors	0	1 (1.4%)	1.0

*1VD* one-vessel coronary disease, *2VD* two-vessel coronary disease, *3VD* three-vessel coronary disease, *DM* diabetes mellitus, *ACEI* angiotensin-converting enzyme inhibitor, *ACS* acute coronary syndrome, *Af* atrial fibrillation, *ARB* angiotensin II receptor blocker, *ARNI* angiotensin receptor-neprilysin inhibitor, *BMI* body mass index = body weight (kg)/height^2^ (m), *CABG* coronary artery bypass graft surgery, *CRT* cardiac resynchronization therapy, *DM* diabetes mellitus, *EF* left ventricular ejection fraction, *eGFR* estimated glomerular filtration rate, *FH* familial hypercholesterolemia, *HF* heart failure, *HT* hypertension, *ICD* implantable cardioverter-defibrillator, *LDL-C* low-density lipoprotein cholesterol, *PAD* peripheral vascular disease, *PCI* percutaneous coronary intervention, *PCSK9 inhibitor* proprotein convertase subtilisin/kexin type 9 inhibitor, *statin* HMG-Co reductase inhibitor.

### Demographic data in subjects with CAD and EF < 40%, who died or survived during the follow-up period

After a median follow-up duration of 44 months, 119 patients had died (Table 3). Compared to the survival group, this death group was older in age, with more having DM (Table 3). The death group also had a lower EF (Table 3). The death group had a significantly lower eGFR (Table 3). There was no difference in terms of the ratio of carriers of FH-related genetic variants between the death and survival groups (Table 3). Regarding clinical history, the mortality group had significantly high proportions of patients with documented peripheral arterial disease (PAD) and ischemic stroke (Table 3). The revascularization history of CABG or PCI was similar between death and survival groups. For medication history, the mortality group had a lower user rate of beta blocker and angiotensin receptor-neprilysin inhibitor (ARNI) (Table 3).

**Table 3:** Demographic data in subjects with coronary artery disease and a reduced ejection fraction (< 40%) (N = 256), who died or survived during follow-up (lipid data at the index admission).

	Death (N = 119)	Survival (N = 137)	*p* value
Age (years)	71.2 ± 11.7	61.2 ± 12.6	< 0.001
Gender (M/F)	95/24	118/19	0.185
BMI (kg/m^2^)	24.9 ± 4.2	25.5 ± 3.8	0.220
Currently smoking, N (%)	15 (12.6%)	27 (19.7%)	0.132
DM N (%)	70 (58.8%)	57 (41.6%)	0.008
HT N (%)	90 (75.6%)	115 (83.9%)	0.117
SBP (mmHg)	126 ± 23	126 ± 24	0.918
DBP (mmHg)	73 ± 14	78 ± 15	0.004
Triglyceride (mg/dl)	125 ± 121	142 ± 107	0.264
Cholesterol (mg/dl)	154 ± 35	160 ± 40	0.296
LDL-C (mg/dl)	94 ± 28	97 ± 33	0.450
eGFR (ml/min/1.73 m^2^)	49 ± 32	67 ± 30	< 0.001
EF (%)	29.1 ± 6.9	30.7 ± 6.3	0.056
TRPG (mmHg)	38 ± 16	34 ± 14	0.068
History			
ACS N (%)	49 (41.2%)	68 (49.6%)	0.209
Ischemic stroke N (%)	28 (23.5)	14 (10.2)	0.006
HF admission N (%)	87 (73.1%)	87 (63.5%)	0.109
Af N (%)	29 (24.4%)	23 (16.8%)	0.161
PAD N (%)	35 (29.4%)	16 (11.7%)	< 0.001
ICD N (%)	3 (2.5%)	4 (2.9%)	1.0
CRT N (%)	7 (5.9%)	4 (2.9%)	0.356
Disease vessel number			
1-VD N (%)	30 (25.2%)	30 (21.9%)	0.784
2-VD N (%)	31 (26.1%)	31 (26.1%)	
3-VD N (%)	58 (48.7%)	72 (52.6%)	
Revascularization			
PCI N (%)	88 (73.9%)	108 (78.8%)	0.378
CABG N (%)	36 (30.3%)	41 (29.9%)	1.0
Medication			
Statin	64 (53.8%)	77 (56.2%)	0.707
ACEI/ARB N (%)	93 (78.2%)	110 (80.3%)	0.757
Beta blocker N (%)	59 (49.6%)	100 (73.0%)	< 0.001
ARNI N (%)	9 (7.6%)	29 (21.2%)	0.003
Ivabradine N (%)	12 (10.1%)	24 (17.5%)	0.105
Antiplatelet N (%)	113 (95.0%)	131 (95.6%)	1.0
PCSK9 inhibitors N (%)	1 (0.8%)	0 (0%)	0.465
FH genetic variant carrier N (%)	7 (5.9%)	5 (3.6%)	0.555

Continuous variables were expressed as mean ± standard deviation.

*ACEI* angiotensin-converting enzyme inhibitor, *ACS* acute coronary syndrome, *Af* atrial fibrillation, *ARB* angiotensin II receptor blocker, *ARNI* angiotensin receptor-neprilysin inhibitor, *BMI* body mass index = body weight (kg)/height^2^ (m), *CABG* coronary artery bypass graft surgery, *CRT* cardiac resynchronization therapy, *DBP* diastolic blood pressure, *DM* diabetes mellitus, *EF* left ventricular ejection fraction, *eGFR* estimated glomerular filtration rate, *FH* familial hypercholesterolemia, *HF* heart failure, *HT* hypertension, *ICD* implantable cardioverter- defibrillator, *LDL-C* low-density lipoprotein cholesterol, *PAD* peripheral vascular disease, *PCI* percutaneous coronary intervention, *PCSK9 inhibitor* proprotein convertase subtilisin/kexin type 9 inhibitor, *SBP* systolic blood pressure, *statin* HMG-Co reductase inhibitor, *TRPG* tricuspid valve regurgitation, peak systolic pressure gradient.

### Baseline demographic data in subjects with CAD and EF < 40%, who had cardiovascular mortality or not during the follow-up period

After a median follow-up duration of 44 months, 68 patients (28.6% of study cohort, 57.1% of the all-cause mortality) had cardiovascular mortality (Table 4). Those who died from cardiovascular causes were older with lower EF (Table 4). Systolic and diastolic blood pressure and total cholesterol were also lower in the group with cardiovascular mortality. Those who died from cardiovascular causes had a borderline lower eGFR (p = 0.053, Table 4). Regarding the clinical history, the cardiovascular mortality group had significantly high proportions of patients with documented atrial fibrillation (Af) and ischemic stroke (Table 4). For medication history, the cardiovascular mortality group had a lower beta-blocker user rate (Table 4).

**Table 4:** Demographic data in subjects with coronary artery disease and a reduced ejection fraction (< 40%) (N = 256), who had cardiovascular mortality (N = 68) or not during follow-up.

	Cardiovascular mortality (N = 68)	Not (N = 188)	*p* value
Age (years)	70.5 ± 11.4	64.2 ± 13.4	0.001
Gender (M/F)	54/14	159/29	0.347
BMI (kg/m^2^)	24.9 ± 3.8	25.3 ± 4.1	0.464
Currently smoking, N (%)	8 (11.8%)	34 (18.1%)	0.257
DM N (%)	38 (55.9%)	89 (47.3%)	0.259
HT N (%)	51 (75.0%)	154 (81.9%)	0.221
SBP (mmHg)	121 ± 19	128 ± 24	0.020
DBP (mmHg)	70 ± 11	77 ± 15	< 0.001
Triglyceride (mg/dl)	134 ± 142	134 ± 102	0.982
Cholesterol (mg/dl)	148 ± 32	160 ± 39	0.030
LDL-C (mg/dl)	92 ± 28	96 ± 32	0.387
eGFR (ml/min/1.73 m^2^)	52 ± 29	61 ± 33	0.053
EF (%)	28.5 ± 7.1	30.4 ± 6.4	0.038
TRPG (mmHg)	41 ± 17	34 ± 14	0.002
History			
ACS N (%)	30 (44.1%)	87 (46.3%)	0.778
Ischemic stroke N (%)	17 (25.0%)	25 (13.3%)	0.035
HF admission N (%)	50 (73.5%)	124 (66.0%)	0.290
Af N (%)	24 (35.3%)	28 (14.9%)	0.001
PAD N (%)	19 (27.9%)	32 (17.0%)	0.075
ICD N (%)	1 (1.5%)	6 (3.2%)	0.679
CRT N (%)	5 (7.4%)	6 (3.2%)	0.167
Disease vessel number			
1-VD N (%)	17 (25.0%)	43 (22.9%)	0.772
2-VD N (%)	19 (27.9%)	47 (25.0%)	
3-VD N (%)	32 (47.1%)	98 (52.1%)	
Revascularization			
PCI N (%)	50 (73.5%)	146 (77.7%)	0.507
CABG N (%)	20 (29.4%)	57 (30.3%)	1.0
Medication			
Statin N (%)	38 (55.9%)	104 (55.3%)	1.0
ACEI/ARB N (%)	52 (76.5%)	151 (80.3%)	0.490
Beta blocker N (%)	33 (48.5%)	126 (67.0%)	0.009
ARNI N (%)	7 (10.3%)	31 (16.5%)	0.240
Ivabradine N (%)	8 (11.8%)	28 (14.9%)	0.684
Antiplatelet N (%)	63 (92.6%)	181 (96.3%)	0.312
PCSK9 inhibitors N (%)	1 (1.5%)	0 (0%)	0.266

Continuous variables were expressed as mean ± standard deviation.

*ACEI* angiotensin-converting enzyme inhibitor, *ACS* acute coronary syndrome, *Af* atrial fibrillation, *ARB* angiotensin II receptor blocker, *ARNI* angiotensin receptor-neprilysin inhibitor, *BMI* body mass index = body weight (kg)/height^2^ (m), *CABG* coronary artery bypass graft surgery, *CRT* cardiac resynchronization therapy, *DBP* diastolic blood pressure, *DM* diabetes mellitus, *EF* left ventricular ejection fraction, *eGFR* estimated glomerular filtration rate, *FH* familial hypercholesterolemia, *HF* heart failure, *HT* hypertension, *ICD* implantable cardioverter- defibrillator, *LDL-C* low-density lipoprotein cholesterol, *PAD* peripheral vascular disease, *PCI* percutaneous coronary intervention, *PCSK9 inhibitor* proprotein convertase subtilisin/kexin type 9 inhibitor, *SBP* systolic blood pressure, *statin* HMG-Co reductase inhibitor, *TRPG* tricuspid valve regurgitation, peak systolic pressure gradient.

### Factors related to all-cause mortality in CAD with reduced EF

Cox regression survival analysis was performed to evaluate the associated factors for all-cause mortality. Carrying the FH-realted genetic variant did not have a significant impact on the survival of CAD with reduced EF (HR 1.228, p = 0.605) (Table 5). Older age had worse mortality. Higher eGFR, higher EF, and beta blocker use were protective with lower all-cause mortality (Table 5).

**Table 5:** Cox regression analyses of associated factors for all-cause mortality in subjects with coronary artery disease and reduced ejection fraction.

Factors	*p* value	HR	95% CI	
			Lower limit	Upper limit
diastolic blood pressure (mmHg)	0.087	0.988	0.974	1.002
Age (years)	0.003	1.028	1.010	1.046
eGFR (ml/min/1.73 m^2^)	0.001	0.987	0.980	0.995
PAD (with vs. without)	0.240	1.286	0.846	1.957
Ischemic stroke (with vs. without)	0.100	1.445	0.932	2.239
DM (with vs. without)	0.089	1.390	0.951	2.031
EF (%)	0.007	0.024	0.002	0.359
FH (genetic variant carrier vs. non-carrier)	0.605	1.228	0.563	2.679
Beta blocker (user vs. non-user)	0.029	0.666	0.462	0.959

Dependent variable: all-cause mortality.

*CI* confidence interval, *DM* diabetes mellitus, *EF* left ventricular ejection fraction, *eGFR* estimated glomerular filtration rate, *FH* familial hypercholesterolemia, *HR* hazard ratio, *PAD* peripheral arterial disease.

### Factors related to cardiovascular mortality in CAD with reduced EF

Sixty-eight patients (26.6% of study cohort, 57.1% of the all-cause mortality) had cardiovascular mortality. Carrying the FH-related genetic variant did not have a significant impact on the cardiovascular mortality of CAD with reduced EF (HR 1.241, p = 0.680) (Table 6). Higher eGFR, higher diastolic blood pressure, and higher EF were protective with lower cardiovascular mortality (Table 6).

**Table 6:** Cox regression analyses of associated factors for cardiovascular mortality in subjects with coronary artery disease and reduced ejection fraction.

Factors	*p* value	HR	95% CI	
			Lower limit	Upper limit
Diastolic blood pressure (mmHg)	0.005	0.972	0.953	0.991
Age (years)	0.275	1.013	0.990	1.037
eGFR (ml/min/1.73 m^2^)	0.021	0.988	0.978	0.998
Af (with vs. without)	0.065	1.659	0.969	2.841
Ischemic stroke (with vs. without)	0.227	1.419	0.805	2.501
DM (with vs. without)	0.310	1.291	0.789	2.111
EF (%)	0.010	0.009	0.000	0.326
FH (genetic variant carrier vs. non-carrier)	0.680	1.241	0.445	3.457
Beta blocker (user vs. non-user)	0.070	0.634	0.387	1.038

Dependent variable: cardiovascular mortality.

*Af* atrial fibrillation, *CI* confidence interval, *DM* diabetes mellitus, *EF* left ventricular ejection fraction, *eGFR* estimated glomerular filtration rate, *FH* familial hypercholesterolemia, *HR* hazard ratio.

## Discussion

Several previous studies investigated the prevalence of genetically diagnosed FH in different clinical scenarios, such as population-based, acute coronary syndrome, premature CAD, or clinically suspected FH cohorts. Here, we genetically screened the prevalence and investigated the prognosis in subjects with CAD and reduced EF in a hospital catheterization-based cohort. Our main findings were the following: A 4.7% prevalence of carriers of FH-related genetic variants among subjects with CAD and reduced EF but carrying the FH-related genetic variant did not have significant impact on all-cause or cardiovascular mortality. However, a higher eGFR and a higher EF had significant protection for reducing all-cause and cardiovascular mortality.

Regarding the prevalence of FH-related genetic variant, the carrier rate is higher in specific clinical scenarios, such as clinically suspected FH, premature CAD, acute coronary syndrome, and serum LDL-C > 190 mg/dL^5,13,15,16,25,26^. The status of carrying the FH-related genetic variant also results in higher adverse cardiovascular events compared to controls with similar lipid profiles^26^. The SAFEHEART registry reported a 3.1-fold increased risk of acute myocardial infarction in the FH + group compared to unaffected relatives^17^. Carrying FH-related genetic variants also leads to early echocardiography-proved left ventricular systolic and diastolic dysfunctions^27–29^. Our study investigated a high-risk condition of angiographic proven CAD with reduced EF and detected a 4.7% genetic FH ( +). However, carrying FH genetic variants had no significant impact on all-cause mortality.

In this study, we totally identified 151 variants, including 6 pathogenic or likely pathogenic variants (in 12 patients, Table 1). The most prevalent variant was *LDLR* c.1747C > T existing in 5 patients in this study cohort. This pathogenic variant was also the most prevalent in our previous study cohort of LDL-C ≧ 160 mg/dL with admission history for coronary angiogram^10^. The *LDLR* c.1747C > T also ranked third in prevalence of FH-related genetic variant in Han Chinese^7^.

Among the VUS, one subject carried the variant of *PCSK9* missense with c.658G > A resulting in a change of p. (Ala220Thr) (classified as VUS by ClinVar). Clinically, this patient had acute myocardial infarction and elevated LDL-C. This variant was also reported in another patient with LDL-C 216 mg/dL and triple vessel coronary disease in our previous study cohort^10^ and two patients in the other familial hypercholesterolemia cohort^30^. Further studies are needed to investigate the functional change at the protein level relating to this *PCSK9* missense variant.

The *APOB* variants that affect the LDL receptor binding domain of apolipoprotein B100 might cause defective binding of circulating LDL-C to LDL receptor of hepatocyte. This type of FH is also known as familial defective apo B100^31,32^, which generally causes a less severe phenotype of FH than *LDLR* mutations^32^. In contrast, familial hypobetalipoproteinemia (FHBL) is mainly caused by protein-truncating variants in the *APOB* gene, resulting in reduced production of apolipoprotein B100 and its assembly with triglyceride, cholesterol and lipoprotein, causing a very low secretion of cholesterol from hepatocyte into blood circulation^33^. In this study, we reported one case with *APOB* c.35_39 del variant, whose LDL-C level was 120 mg/dl status after high-potency statin treatment (Table 1). This variant was classified as pathogenic for FH in ClinVar^34^ and one study listed this variant as a cause of monogenic FH^35^. However, no functional study for this variant is reported yet^34^. Further studies for this *APOB* variant are needed for its downstream effect on protein functional change and to clarify whether it can cause FH or FHBL.

Regarding the prognostic factors for HF with reduced EF, previous studies have shown that chronic kidney disease and lower eGFR were significantly associated with worse survival^36,37^. Impaired renal function (eGFR < 60 ml/min/1.73 m2) on admission independently predicts long-term mortality in patients hospitalized for HF, regardless of HF phenotypes^36^. Furthermore, worsening renal function within one year is strongly associated with increased mortality in patients with HF and reduced EF^38^. Our study corroborated the detrimental impact of poorer renal function on the survival of CAD with reduced EF.

The use of beta blockers was protective for survival in HF with reduced EF or post-myocardial infarction status^39,40^. The updated heart failure guideline recommended a class 1A indication for the use of beta blockers in heart failure with reduced EF^41^. However, in real world clinical practice, beta blocker was frequently under-prescribed. Our study re-iterated the importance of beta blocker use for protecting survival in CAD with reduced EF.

There are some limitations of our present study. First, this is a single hospital catheterization laboratory cohort. Therefore, there was potential selection bias, and the case number was limited, thus lacking enough power for a genetic study. Second, we did not investigate mutations of *APOE* polymorphism, especially the frequency of E4 allele^42^ and autosomal recessive mutations in *LDLRAP1*^43^ or *STAP1*^4,44^. Third, we did not further discriminate the specific type of pathogenic variant and its severity (that is, defective LDLR versus null receptor) and to compare their prognostic impacts^4,25^. Fourth, the ClinVar database is dynamic, today’s VUS or “likely benign” may switch to “likely pathogenic” or “pathogenic” in the future. Fifth, we lacked protein functional data for *APOB* c.35_39 del variant to clarify whether it can cause FH or FHBL. Moreover, some of FH patients did not have a traceable baseline LDL-C data before statin use for realizing the impact of FH on their lipid profiles.

In conclusion, the prevalence of carriers of FH-related genetic variants in our hospital catheterization-laboratory based cohort of subjects with CAD and reduced EF was 4.7%. Carrying the FH-related genetic variant had no significant impact on survival. However, higher GFR, better EF, and beta blocker use had protective impacts on survival in patients with CAD and reduced EF. Further larger study is needed for evaluating the impact of carrying the FH-related genetic variant on the survival of CAD and reduced EF.

## Data Availability

The datasets generated and/or analyzed during the current study are not publicly available because the personal identification data were not anonymous or pseudonymized but are available from the corresponding author on reasonable request.

## Abbreviations

1VD: One-vessel coronary disease
2VD: Two-vessel coronary disease
3VD: Three-vessel coronary disease
ACEI: Angiotensin-converting enzyme inhibitor
ACS: Acute coronary syndrome
ACMG: The American College of Medical Genetics and Genomics
Af: Atrial fibrillation
APOB: Apolipoprotein B
ARB: Angiotensin II receptor blocker
ARNI: Angiotensin receptor-neprilysin inhibitor
BMI: Body mass index
CABG: Coronary artery bypass graft
CAD: Coronary artery disease
CI: Confidence interval
CRT: Cardiac resynchronization therapy
DBP: Diastolic blood pressure
DM: Diabetes mellitus
EF: Left ventricular ejection fraction
eGFR: Estimated glomerular filtration rate
FH: Familial hypercholesterolemia
FHBL: Familial hypobetalipoproteinemia
HDL-C: High-density lipoprotein cholesterol
Het: Heterozygous
HF: Heart failure
HR: Hazard ratio
HT: Hypertension
ICD: Implantable cardioverter-defibrillator
LDL-C: Low-density lipoprotein cholesterol
LDLR: Low-density lipoprotein receptor
MDRD: Modification of diet in renal disease
NGS: Next generation sequencing
PAD: Peripheral vascular disease
PCI: Percutaneous coronary intervention
PCR: Polymerase chain reactions
PCSK9: Proprotein convertase subtilisin/kexin type 9
SBP: Systolic blood pressure
SNP: Single nucleotide polymorphism
Statin: HMG-Co reductase inhibitor
TRPG: Tricuspid valve regurgitation, peak systolic pressure gradient
